# 100 years of mathematical cosmology: Models, theories and problems, Part B

**DOI:** 10.1098/rsta.2021.0171

**Published:** 2022-08-22

**Authors:** Spiros Cotsakis, Alexander P. Yefremov

**Affiliations:** ^1^ Institute of Gravitation and Cosmology, RUDN University, ul. Miklukho-Maklaya 6, Moscow 117198, Russia; ^2^ Research Laboratory of Geometry, Dynamical Systems and Cosmology, University of the Aegean, Karlovassi 83200, Samos, Greece

**Keywords:** universes, mathematical models, evolution of ideas, introductory survey

## Abstract

We continue our overview of mathematical cosmology with a survey of the third and fourth periods of the development of the subject. The first Part includes the first two periods and is published separately. The third period (1980–2000) continues here with brief descriptions of the main ideas of inflation, the multiverse, quantum, Kaluza–Klein, and string cosmologies, wormholes and baby universes, cosmological stability and modified gravity. The last period, which ends today, includes various more advanced topics such as M-theoretic cosmology, braneworlds, the landscape, topological issues, the measure problem, genericity, dynamical singularities and dark energy. We emphasize certain threads that run throughout the whole period of development of theoretical cosmology and underline their importance in the overall structure of the field. We end this outline with an inclusion of the abstracts of all papers contributed to the second part of the *Philosophical Transactions of the Royal Society A*, theme issue ‘The future of mathematical cosmology’.

This article is part of the theme issue ‘The future of mathematical cosmology, Volume 2’.

## 1. Introduction

This is the second part of our introductory survey of mathematical cosmology. The first part is contained in volume 1 of the Theme Issue ‘100 Years of mathematical cosmology’ and is separately published covering the period of development of mathematical cosmology starting from 1917 until 1980. In this second part, we cover the period since 1980, and provide brief descriptions of the emergence, development, importance and interconnections of most major subfields of theoretical mathematical cosmology, such as modified gravity and dark energy, inflationary, quantum, string, M-theoretic and brane cosmologies, wormholes, measures, stability, genericity and topology. Here, as in Part A, we focus on key theoretical discoveries as well as fundamental ideas that were to become (or, in fact, may become) instrumental in the development of the whole field. At the end of this paper, we include the contents of the individual invited contributions to volume 2 of the Theme Issue ‘The future of mathematical cosmology’.

## 2. Third period, 1980–2000

In this section, we discuss the following novel ideas that emerged in this period:

— Inflationary cosmology
— The multiverse
— Wave function of the universe
— The cosmological measure problem
— Baby universes and wormholes
— Kaluza–Klein universes
— String cosmology
— f(R) gravity and cosmology
— The issue of cosmological stability

### (a) Inflation

In the late 1970s, dramatic discoveries in particle physics like asymptotic freedom and the various issues and approaches of how vacuum fluctuations may lead to models of grand unified theories of electromagnetic, weak and strong interactions, completely changed that field, and it was not long before the consequences of these developments for ‘early universe’ cosmology were followed. Particle physicists found in cosmology a testing ground for their theories, while cosmologists realized the power of high-energy arguments for the complex problems that they were facing at the time. *Particle cosmology* was thus born because of the need to better understand the behaviour of matter and fields in the extreme conditions of the big bang.

*Inflationary cosmology* [1,–3], a paradigmatic application of particle physics ideas to cosmology. Unlike more ground-to-earth applications of nuclear physics and elementary particle theory to cosmology, like big-bang nucleosynthesis and baryogenesis, the appearance of inflation was a truly exotic idea, but at the same time had some attractiveness to many cosmologists who had a good background of general relativity. It was based on the transient effects of a scalar field for an accelerated expansion of the universe (in de Sitter’s and in Lemaître’s models discussed earlier, acceleration appears as a *permanent* property, not a transient one).

At a proper time hypersurface $t∼10^{-35}\;s$, we imagine that the particles comprising the material content and occupying different vacua of different energies were displaced from their equilibrium states and were able to move to other vacua of lower energies. During this motion between initial and final vacuum states occupied by matter, the vacuum energy liberated creates a gravitational repulsive force similar to the original Einstein’s cosmological constant and so the universe accelerates for the brief period between the two, the old and the new, vacuum states.

This is very important. For each such pair of vacua, particles arriving at new vacua are able to form ‘bubbles of new vacua’ nucleating at different spatial regions in different times. According to Guth, this phase transition supercools the universe by 28 or more orders of magnitude below a critical temperature, and then leads to a huge expansion of exponential growth accompanied by a disastrous process of all sorts of irregularities in the curvatures and the matter density arising in this phase of evolution of the universe. (This was ameliorated, however, in subsequent models by other authors making the motion between vacua *slower*-leading to a unique bubble comprising the visible universe.) In fact, a slight change in the original scenario of Guth’s showed that in the ‘new inflation’ scenario (as in various others - see below), inflation is in fact eternal [4].

But the inflationary idea, common to all inflationary models that followed Guth’s, also leads to a much larger, homogeneous and isotropic universe, with many of the puzzling issues of the previous models practically solved. That includes the horizon, flatness, monopole, as well as other perplexing issues, unexplained in the standard model of cosmology. For instance, the visible universe today—our own bubble—comprised a huge number of causally disconnected regions at Planck time (and also at the time of inflation). But during inflation one of these regions was inflated to encompass all others, allowing thus a restored type of causal communication between them and leading to today’s observed uniformity.

In fact, it is a most important property of the resulting inflationary picture of the early universe, also improved and generalized by various later modifications or extensions, that by the same mechanism it also explains other additional properties: the temperature and energy-density fluctuations of the cosmic microwave background radiation as a result of the quantum phase during the inflationary stage, namely, the quantum fluctuations of the scalar field driving inflation (see [5] for background in the theory of cosmological fluctuations, [6] for an early review of the inflationary approach to this problem, [7] for the situation in the early 1990s and [8] for a modern review of this idea).

Today the development of the inflationary picture of the early universe continues. There are practically hundreds of model-dependent implementations of inflation in different theories, for instance in relativity, modified gravity, supergravity, superstring cosmology, M-theoretic cosmology, etc., but there are two issues of importance related to the inflationary scenario that require attention, and which show that inflation is certainly not the last word about the structure of the early universe.

The first is to test how successful inflation models fit cosmological data coming from *observations* made by various satellites and instruments, COBE, PLANCK, WMAP BICEP/KECK, etc. Indeed, many of the most popular inflationary models are now definitely falsified by these data. This is made possible by the consideration of the slow-roll parameters ϵ and η defined by the scalar field potential and its first and second derivatives. Eventually, the predictions of inflationary models are measured by certain combinations of the slow-roll parameters calculated in the models, the primordial tilt $n_{s}$ and the tensor-to-scalar ratio r. These are functions of the number N of the inflationary e-folds, which occur between the time when a given perturbation leaves the horizon and the end of the inflationary period, and describe quantum fluctuations induced by inflation such as the gravitational wave energy spectrum. Interestingly, current observations give a value of r<0.036 at 95% confidence [9], too low for most inflationary models to predict.

The second issue is what would be a believable *prequel to the inflationary stage* that would make the inflationary idea more natural. We shall come back to this issue in subsequent sections of this paper.

### (b) Multiverse

Even though according to inflation the bubble leading to our visible domain adequately expanded and inflated solving in this way many of the cosmological conundrums of standard cosmology, the complex process of creation and evolution of the whole network of such distinct inflating bubbles may lead to a very awkward situation, now elaborated to what is called the *multiverse*.

The fact that inflation can be *eternal* was realized soon after Guth’s original idea, by Paul Steinhardt [4] and by Alexander Vilenkin [10] for new inflation, and later by Andrei Linde [11] in the context of the chaotic inflation scenario, where the potential of the scalar field driving inflation has no flat plateaux. Linde used heuristic arguments to imply that the process of large-scale fluctuations of the scalar field leads to an *eternal*, *self-reproducing*, *inflationary multiverse*. In this, each inflating bubble consists of further inflating subregions with each one of these in turn having further similar ones, at infinitum, a process seemingly progressing endlessly to the future as well to the past. In this multiverse, the density parameter Ω varies continuously between 0 and 1, so our bubble together with infinitely others with Ω∈(0.2,0.3) lies somewhere in the structure with non-zero probability.

It is a difficult problem how to calculate probabilities in the multiverse [12], and in a situation like this one may produce arguments leading to an overall controversial picture (cf. e.g. [13] and references therein) for the probability of the occurrence of a stage of inflation. For instance, in [14,15] one finds opposite views and probabilities ranging from almost one to almost zero. Despite being hard to believe, this picture appears nevertheless as a natural extension within the wider context and philosophy of inflationary cosmology, and some recent progress has indeed been made (see §3c). In fact, most authors agree that inflation is future-eternal [16], but probably not being so in the past [17] without violating the null energy condition [18].

### (c) Quantum cosmology

For the consideration of the *initial state of the universe* as a quantum problem, the so-called programme of *quantum cosmology*, one such approach is through the *Wheeler–DeWitt equation* advocated by DeWitt [19] and Wheeler [20], describing the evolution of the quantum wave function of the universe considered as a quantum object. Solutions of the Wheeler–DeWitt equation then give probabilities for the universe to evolve from one state to another, provided one gives prescribes how to specify ‘initially’ both the probability amplitude and its first derivative normal to the initial hypersurface in superspace (this is a set describing the space of all 3-geometries). One approach to the study of this equation was to consider the limiting case of ‘minisuperspace’, a subset of the full problem where most of the gravitational degrees of freedom are frozen out. This turns the whole quantum cosmology problem, considered in ‘canonical quantum gravity’, into a more manageable quantum mechanics problem, and many different models have been considered in this framework.

A prequel theory to inflation remains a very mysterious problem and its solution is largely unknown even today. This is perhaps one of the most important ingredients necessary to make our current (and future) cosmological standard models more complete. In a sense, initial or boundary conditions are necessary for any cosmological model to be more predictive. All these issues became more substantial and received a blow of original ideas in the 1980s (the decade 1980–1990 may be called indeed a ‘golden decade’ in theoretical cosmology research).

In 1983, Hartle & Hawking introduced a radical approach using path integral methods to describe the problem of initial conditions in quantum cosmology introduced in the 1960s [21]. That approach, called the *no-boundary proposal* for the wave function of the universe, suggested a beginning of the universe not as dramatic as that appearing in the standard model of the universe. In fact, there is a beginning but the initial singularity is now replaced by a situation where time is imaginary, there is a smooth passage to a quantum regime, a creation ‘from nothing’. The *Hartle–Hawking wave function* also peaks as most probably states those that describe infinitely large and empty universes.

However, it soon became clear that other, equally probable and similarly constructed, ‘creation-from-nothing’ proposals are possible, but with completely different predictions. For instance, a small, hot early universe, pretty much like the standard big bang model of the classical universe, is the prediction of *A. Vilenkin’s tunnelling model* creation-from-nothing 1985 model [22], which also predicts a period of exponential expansion [23].

Soon however, the problem arose of what it means for the universe to be in a *typical quantum state*, and what is the meaning probability in quantum cosmology as well as the possible set of all these different wave functions (see [24] for a clear discussion of the difficulties in defining a probability of inflation in a simple minisuperspace model). This is a very deep problem and is related to the measure problem in cosmology and the issue of how to define probabilities in a cosmological setting (see the next section).

The first works resulted in a huge proliferation of very interesting subsequent papers on quantum cosmology during the 1980s (see [25] for a bibliography of papers for that period), addressing an impressive variety of very deep questions and meaning in cosmology (prediction, time, creation, etc.).

Finally, we mention a very different approach to quantum cosmology, *loop quantum cosmology* (LQC). This is based on an approach to the quantization of gravity using new Hamiltonian variables (the Ashtekar variables), and taking seriously the Dirac quantization method of field theory. LQC also uses a different set of connections than the Levi–Civita one, the spin connection, and this results in an improved behaviour of the original Wheeler–De Witt equation. This also gives a bounce instead of the initial singularity in FRWL models considered in LQC, whereas many other cosmological properties acquire a new light in this framework. For reviews, see [26,27].

### (d) Measure-theoretic cosmology

There are many situations in theoretical and mathematical cosmology where one is interested in ‘typical behaviour’, the ‘most probable state’ a cosmological system may assume, in short, one is interested in a measure of the ‘degree of genericity of a given property’. For example, in inflationary cosmology, there is the question of how typical is an inflationary stage within a given set of solutions. For instance, consider a flat FRWL model with a perfect fluid with equation of state p=wρ, where p,ρ are the pressure and the density of the fluid, respectively. Then, for different values of the parameter w, one obtains different dynamical regimes described by exact solutions for given w values, and the question arises as to what is the long-time behaviour of the solutions of the system. For a scalar field potential quadratic in the field, it may be shown [28] that inflationary stages are an unavoidable property of most solutions of the associated dynamical system, and in addition for exponential potentials, power-law inflationary stages are likewise a stable attractor of the system [29].

To study probabilities in a classical or quantum context in cosmology more generally, it is necessary to have a suitable measure in the space of all classical solutions of the theory, much like the Liouville measure (i.e. the volume element) in statistical physics.

Such a measure was first proposed by Henneaux [30] and by Gibbons *et al*. [31], and we shall refer to it as the *HGHS canonical measure* on the set of all universes. This measure plays the role of a Liouville-type measure in a minisuperspace approximation of the cosmological phase space, and its construction assumes a Hamiltonian flow and associated symplectic structure and constitutes an adaptation of the symplectic quotient construction in cosmological problems.

The application of the HGHS measure to cosmology, most importantly perhaps to the structure of the multiverse, is, however, plagued with various infinities. In addition, there are various conflicting claims in the literature for inflationary versus non-inflationary solutions; in many cases of interest such as a massive scalar field [32], $R^{2}$ inflation [33] and Bianchi-I inflation [34], the HGHS measure gives infinite answers on both types of solution. In [35] (see also [36]), however, a cut-off in the dynamics is introduced, for which the resulting measure becomes finite because the probability of N e-folds of inflation is then exponentially suppressed, thus making inflation improbable.

Other studies of the measure such as [37] point out related difficulties associated with the HGHS measure, such as the use of minisuperspace approximations, the lack of inhomogeneity considerations, as well as the lack of any interaction between different pocket universes in the ensemble to which the measure is applied (so as to allow for ‘equilibration’ on the relevant scales), while a comparison between different measures can be found in [38]. A broader perspective on measures in the multiverse is found in [39].

Despite all these extremely valuable works, it is indeed true that the measure problem is currently unresolved and more research is clearly needed in this most interesting problem of mathematical cosmology.

### (e) Wormholes and baby universes

Wormholes connect parts of an asymptotically flat (Euclidean) region in space–time, or perhaps two different asymptotically flat regions, and are important because according to Hawking describe possible quantum states of closed universes that branch off from our own [40]. One may imagine wormholes as small tubes connecting various types of two closed 3-boundaries, or ‘baby universes’, the latter being closed carry zero energy and momenta. (The word ‘semi-wormhole’ is sometimes used to designate a tube that connects a state with no baby universe to another with one.)

The importance of wormhole-like objects in quantum cosmology took off in the late 1980s when Coleman showed how they can be used to make the cosmological constant vanish [41]. For this purpose, he used the Hartle–Hawking wave function to show that it had a peak at configurations for which the cosmological constant vanished. In this situation, λ is contained in the leading term in an expansion of the classical action, while all other higher-order terms (i.e. $-R+aR^{2}+b{\text{Ric}}^{2}+c{\text{Riem}}^{2}+\cdots$, with the coefficients depending on the wormhole being summed) vanish, being the saddle points of the action, i.e. Einstein spaces. Then the effective action Γ is found to be simply inversely proportional to λ, hence peaked at λ=0.

This spectacular result was criticized because it was thought that it led to a situation having a network of large wormholes on very large scales with very high density (of the wormhole ends in the dominant Euclidean field configurations)—a catastrophic prediction of strong, non-local interactions [42]. Nevertheless, it led to a very large number of works on the varied physical effects of wormholes (see, for instance, the interesting proceedings at that time [43]).

Another cosmological application of wormholes is in the construction of bouncing models which avoid an initial singularity. It was first shown by Morris and Thorne that wormholes can be solutions to the Einstein equations that violate the null energy condition but allow for time travel [44,45]. Various such wormhole solutions exist with matter models, or various scalar fields, in modified gravity, scalar-tensor theories, or in brane universes, see [46,47]. A particularly interesting wormhole solution that requires no violation of the energy conditions, is a cylindrical wormhole with spherical topology near the throat [48]. Another example of such a configuration is a generalization of the Tolman universe (see the section on the Tolman universe in Part A of this survey). That was a bouncing universe but the nature of the bounce is somewhat ambiguous as it is not precisely specified. Wormhole solutions connecting a previous phase in the evolution of the universe to the current one can provide the missing link, and many such models have been built (see [49] for a textbook presentation). A Tolman wormhole is an example of such a bouncing model with the strong energy condition being violated [50].

### (f) Kaluza–Klein cosmology

An additional, important input to ‘alternative’ cosmology was provided by the multitude of possibilities in the development of *Kaluza–Klein cosmologies* (see [7,51,53] for a concise, almost textbook treatment, while [54] is more suitable for relativists (this paper contains almost 400 references)).

In such a higher-dimensional setting, there are two kinds of spatial dimensions, the usual ‘external’ (large) ones and the internal (small, that is ‘compact’ ones, see below) dimensions, which play a key role in determining the structure of the physical laws in the overall higher-dimensional set-up (extra time-like dimensions are associated with ghost instabilities and are thus less favourable). There is a fundamental ‘cylinder condition’ that defines circle-compactification, meaning that nothing depends on the extra Y coordinate, or equivalently, the space that is defined by the extra coordinate(s) is compact in the subspace topology.

This leads to a very small scale (volume) of the compact dimensions compared with the remaining ones, and so their associated energy must be very high. Since in these models, the Newton constant, which gives the range of the gravitational interaction, appears as inversely proportional to the very small volume of the compact internal dimensions, this led some to propose a higher-dimensional mechanism for explaining why the gravity scale is so much larger than those of all the other interactions, the so-called hierarchy problem (see §3b).

Also the fundamental physical constants that appear in the low-energy theory that we observe have values that crucially depend on integrating over the extra dimensions. The internal dimensions are also static, in distinction to the external ones that are dynamic, usually expanding or contracting, and this situation has led to a great number of works that approach the standard problems of cosmology (nature of singularities, bouncing models, horizon problem, etc.) from such a higher-dimensional perspective (cf. the quoted references for various examples of such models).

One particularly interesting development in this context has been the issue of whether or not chaotic Mixmaster behaviour is possible with more spatial dimensions. After initial results [55,56] about the disappearance of chaotic behaviour in vacuum five- and seven-dimensional Kaluza–Klein universes, respectively, with static internal dimensions, in a series of very interesting papers in the middle 1980s, J. Demaret *et al.* argued that in homogeneous [57] as well as inhomogeneous [58] vacuum Kaluza–Klein cosmologies more dimensions lead to less chaos, in particular, chaos disappears in 11 or more space–time dimensions, but it remains in homogeneous vacuum Kaluza–Klein universes in space–time dimensions between 4 and 10 [59] (the space–time dimension 11 has been called the ‘critical’ dimension for chaotic cosmology in [60]).

But two issues in all such models remained: What is that which makes some dimensions expanding while others are kept small, and second, how does one make the (small) size of the internal dimensions stable with respect to physically relevant perturbations? Both of these problems are addressed in more elaborate extensions of the interesting initial Kaluza–Klein cosmologies of the 1980s.

### (g) String cosmology

A new chapter in mathematical and theoretical cosmology opened with the application and exploitation of the new duality symmetries between string-theoretic models and 11-dimensional supergravity, suggesting the existence of a still larger theory—M-theory—in which gravity being supersymmetric propagates in the 11th dimension while the remaining interactions are constrained on suitable 10-dimensional hypersurfaces. This new set-up requires in addition to the metric, other fields—for instance two more massless states, the dilaton and an antisymmetric tensor field (commonly called ‘the axion’).

For cosmology, all this new information translates into a huge variety of suitable gravitational Lagrangians including all sorts of other fields, leading to solutions which cannot exist in general relativistic or other similar contexts. The result is *string and M-theoretic cosmology*, a new and largely unexplored field of investigation (for excellent textbook introductions, see [61], and the last Part of [62]).

The duality symmetries when applied to a homogeneous Bianchi I cosmology lead to the so-called scale-factor dualities, which not only invert the scale factors but at the same time shift the dilaton, therefore making this a purely string-related effect. When combined to time-reversal symmetries, and applied to a Friedmannian background, one is led to a good result: In *the pre-big-bang model* of Gasperini & Veneziano [63], the singularity has moved to infinity, and its place is taken by a smooth evolution bouncing through it from a previous expanding to a post big bang contracting homogeneous and isotropic state. (More general results are possible in anisotropic contexts.)

The resulting universe-model is characteristically distinct from the hot big bang cosmology, in that the curvature has a maximum at the end of an initial cold, unstable and vacuum state. One may compare the quantum cosmological approaches to the hot big bang versus the string perturbative vacuum state of string cosmology in a quantum setting, where the latter overpasses some of the well-known problems of the standard approach to quantum cosmology, and introduces a tunnelling not ‘from nothing’ but from the initial string state (see [61] for a complete treatment of the various results in this approach). A potential issue with this model is, however, the possible instability of single semi-classical trajectories, an issue which could be bypassed in a path integral approach.

More generally, string cosmological models offer new possibilities for cyclic behaviour (cf. the interesting qualitative work [64] and references therein), exact solutions of Bianchi type made possible with new forms of the antisymmetric H-field [65], and a variety of inhomogeneous solutions with an initial Kasner era [66]. For more results of this type, see the comprehensive review [67].

### (h) f(R)-cosmology

A rather different modification of the basic dynamical form of general relativity than the Brans–Dicke or more generally the scalar–tensor theories—higher-order gravity—appeared very early and continues in new forms until today, having major ties with early universe cosmology. Based on earlier impressive and original work of Lanczos (cf. [68,69] and references therein) and others on the influence of higher-order invariants of the Riemann tensor on the structure of general relativity, a particular choice eventually seemed simple and general enough: the gravitational Lagrangian be an arbitrary analytic function of the scalar curvature, f(R). In fact, this leads under a metric variation to fourth-order field equations, not second order-like general relativity (while other types of variation may lead to second-order ones, see below). The result is a new theory of gravitation, *f(R)-gravity*.

Keeping f(R) analytic means that this type of theory will be expected to play a significant role in the extreme conditions of the *early* universe where higher-powers of the scalar curvature become important, but not for late-time evolution (where such powers become negligible). f(R)-gravity is hence expected to convey some of the importance of the influence of quantum gravitational effects in the early universe even though everything is classical.

Some first impressions of f(R)-gravity to cosmology were given in the period considered (e.g. [70,–73]), but the field was meant to blow up in activity starting in the mid-1980s, and this continues until the present time (see the many reviews of this theory towards the end of this paper).

In 1983 in a seminal paper, Barrow & Ottewill [74] showed that under especially simple algebraic conditions, the whole family of theories in f(R)-gravity allows for inflation, as well as it provides stable FRWL, de Sitter cosmologies with respect to perturbations outside general relativity, that is in f(R) theories. In addition, it was shown a little later that f(R)-gravity is conformally equivalent to Einstein’s theory when another purely cosmological, self-interacting scalar field is added [75].^1^ This equivalence of the two dynamically different theories required the same type of matter as inflation did, but it also showed that f(R)-gravity leads to a symmetric-hyperbolic system, in contradistinction to other types of modified gravity. This equivalence also allowed to transfer a number of results from general relativity to the generalized framework of f(R)-gravity, including the singularity theorems (because conformal transformations respect causality).

With regard to conformal transformations in such contexts, there is an important geometric generalization of general relativity, conformal (Weyl) gravity. This theory has a unique place among all theories quadratic in the curvature invariants, since it is conformally invariant. (It is also closely related through the Gauss-Bonnet theorem to a particular Bach Lagrangian.) This leads to intriguing properties and connections with various important and largely unexplored issues, such as what breaks conformal invariance, and how this is related to a number of cosmological puzzles. For more on this theory cf. [76].

The development of *f(R)-cosmology* continues today at an accelerating pace. Two developments are of particular importance. The alternative Palatini formulation of the f(R)-gravity equations [77] leads to a reduction of order and can therefore lead to simplified treatments of a number of issues in cosmology (for a review of cosmology in Palatini theories, see several of the reviews cited at the end of this paper).

Second, there are new developments of *no-scale* f(R)-theory [78], that is Lagrangian f(R) theories in which the field equations are traceless versions of the standard ones. An important feature of these theories is that conformally they become GR plus a scalar field but with the crucial difference that the self-interacting potential is scale-invariant. This exact result implies different CMB parameter forms for all previous forms of f(R)-gravity. For the traceless version of the quadratic theory, the prediction for the tensor-to-scalar ratio is given by, $r=12/(b^{2}N^{2})$, with b≠1 is the new condition necessarily required in the traceless version [78]. (Here N stands for the e-fold function that measures the length of inflation, while b is the crucial new arbitrary constant related to the scale invariance of the potential when we scale the field in traceless theory.) This theory accommodates any r-value however small.

### (i) Cosmological stability

We mentioned in Part A of this survey that the most important property of the Einstein static universe was its transient character. This is based on its instability, as in the Eddington–Lemaître cosmology. In fact, the issue of the instability of the Einstein static universe is much wider, and has been investigated by different authors who proved instability in various current contexts.

Starting with a radiation-filled Einstein universe, instability was shown in terms of inhomogeneous oscillatory modes [79], or in a fluid-filled model with respect to conformal metric perturbations (where the interesting result is that there are regions of stability depending on the sound speed) [80,81]. Studies of instability were then exploited by Ellis & Maartens to introduce the *emergent universe* [82], where in the context of inflationary cosmology, the Einstein static model serves as a prequel to the inflationary stage: an transient initial state with matter being a scalar field whose vacuum energy determines the cosmological constant of the model. This model has no singularity, no horizon problem and, most importantly, does not need a quantum era in its early evolution, it is past eternal. Its instability of the initial Einstein static state is proved [83], but the effects of other, inhomogeneous, nonlinear perturbations are not yet concluded (see, however, a proof against Bianchi-IX homogeneous perturbations in [84]). Last, the Einstein static universe, perhaps even as a transient model, does not seem to pass the finite action requirement [85].

The issue of cosmological stability is generally related to various geometric properties of the flow of a system of equations. For Bianchi universes in vacuum or filled with various fluids, exact solutions are known and so issues of stability have been studied by many authors (see [86] for a review of the exact solutions in this context). The systems are described by ordinary differential equations but the stability of most types (e.g. those of type V, VI, VII) becomes non-trivial to decide because of the typical appearance of zero eigenvalues in the case of future asymptotic evolution [87].

For Bianchi cosmologies with a positive λ, there is a result of Wald [88] to the effect that all (including B-IX with large enough λ) approach the de Sitter solution, and are therefore isotropized. There were various such results in the 1980s and the 1990s pointing to the validity of a *cosmic no-hair theorem* for such space–times also in extended frameworks [89] such as higher-order gravity [90], or quantum cosmology [91].

Stability really depends on how one sets the problem, and there are various different and inequivalent definitions in the literature. It is closely related to the problem of describing the asymptotic states of a cosmology, and for this there exist various different approaches (see [92,93] for homogeneous cosmologies). This is a vast field within mathematical cosmology, rapidly expanding today. For inhomogeneous models, the situation is far less well understood; see the next section.

## 3. Fourth period, 2000 until today

During the last 20 years in the evolution of mathematical cosmology, one sees an explosive mixture of joint developments in many of the aforementioned areas as well as the formation of many novel ones. It is fair to say that the whole field has matured to a degree beyond recognition as compared with, say, its image 50 years ago. In this last part of the development of the field, we shall focus primarily on the following topics:

— M-theory and cosmology
— Braneworlds
— The landscape
— Topological issues and dynamical evolution
— Genericity in cosmology
— Models of dark energy

### (a) M-theoretic cosmology

It was a surprise to many that the quantum consistency of superstrings eventually required the existence of further (than strings) low-dimensional objects, the p-branes, with p taking ‘small integer values’. Once one allows for strings (that is p=1 objects) into the theory, however, it is completely natural to expect that other such objects play an important role and consider these other possibilities. Branes are ubiquitous and largely unexplored, and given the various dualities between the original five string theories and 11-dimensional supergravity, it is only natural to explore them in this generalized context of interconnected theories coined *M-theory*. In fact, in the *string or brane gas scenario* of Brandenberger *et al.* in 2000 [94], the universe is supposed to be a ‘hot soup’ of branes of all p dimensions, topologies and spatial orientations, and all internal dimensions are assumed compact, in an attempt to explain why only three dimensions are surviving. In the simplest models of this kind, branes of larger dimensionality have higher energies and annihilate first, leaving only strings. Brane cosmology is a very rich subject, and some aspects of it are treated in §3b.

A most important aspect of M-theory cosmology is revealed in the study of chaotic Mixmaster behaviour in these frameworks. Initial results [95] pointed to the disappearance of the BKL chaotic oscillations on approach to the string cosmological singularity in *homogeneous* Mixmaster models, due to the combined effect of both the dilaton and the axion fields. A more elaborate treatment of this problem brings us into the realm of the general effects of p-form fields often coming from supergravity theories and M-theory.

A spectacular effect of these fields was shown to exist in the generic inhomogeneous case of the old Mixmaster universe but now considered in the context of superstring or M-theory cosmologies, where it was shown that the role of the p-form fields is similar to that of the curvature terms in the Einstein theory, namely they act like potential walls, thus preventing the possibility of free Kasner motions of the universe point [96]. The overall result in any space–time dimension in the general inhomogeneous case is again the BKL behaviour, even in the case of 11 or more space–time dimensions where that erratic behaviour was known to be absent in pure Einstein gravity [97]. This led to the interesting speculation of a possible *de-emergence* of a classical or even a quantum behaviour of space–time as we approach the initial singularity in these models, and the possibility of an effective, purely algebraic description of the dynamical behaviour of the universe near the singularity in terms of hyperbolic Kac–Moody algebras [98].

Today, about 20 years since its original conception, M-theoretic cosmology is only just beginning. In a series of interesting papers, Lucas *et al.* [99] (see also [100] and references therein) introduced and studied a general framework for doing cosmology in type I superstring and M-theory, with particular emphasis on the relation between supergravity p-brane solutions and cosmology. Because of an allowed exchange between the time coordinate and the transverse spatial coordinate in these solutions, the intriguing possibility opens to explaining a number of cosmological phenomena through stringy considerations.

For qualitative applications of dynamical systems techniques in the context of an FRWL model with six Ricci flat internal dimensions and the 11th dimension compactified on a circle, see [101]. A feature of M-theory cosmologies appears to be their transient acceleration, see [102] or many related references, and [103] for a more elaborate analysis of this property. An interesting ingredient used in these works is a correspondence between the space of flat FRWL cosmologies and that of geodesics of a suitable target space, with accelerating ones occupying a special subset of the lightcone [104].

For background necessary for string- and M-theoretic techniques particularly adapted for the relativist, see the relevant sections in [61] as well as [105].

### (b) Braneworlds

Braneworlds, or p=3-branes, represent universes (i.e. four-dimensional space–times) lying inside a higher-dimensional space dictated by superstring theory, in which the three particle physics interactions ‘live on the brane’ while gravity propagates freely in all dimensions of space, and is much weaker than the other forces. This is in sharp contrast to the usual Kaluza–Klein cosmology wherein the extra dimensions are necessarily static and presumably compactified, hence unobservable.

Brane theory seriously elaborates further on this very original Kaluza–Klein idea. There is a set of consistency conditions of the quantum superstring theory that leads naturally to the exploration of the possibility of having ‘large extra dimensions’, dimensions that have a compactification scale not at Planckian energy but much lower, possibly comparable with the currently favourable ones (cf. [106,107] and references therein). A concrete realization of these ideas was originally introduced by Randall & Sundrum [108] in 2000, where the Einstein cosmological equations are modified by the addition of extra terms coming from the brane embedding in the bulk space, as well as the bulk space itself.

In the resulting set-up new possibilities are possible for inflation, gravity ‘induced’ on the brane by localizing matter, or more general cosmological dynamics (see [61], ch. 10 for a short review). One may imagine a number of such lower-dimensional universes coexisting in the parent space and moving in one or more of the extra dimensions. Using the mathematics of submanifolds, it is not very difficult to describe the geometric properties of this set-up as well as the modified Einstein equations, cf. [109,110].

In fact, there are several scenarios in the literature about possible brane models of the early universe, a particularly interesting one being the *ekpyrotic universe* proposed in 2001 by Khoury *et al*. [111] (see [112] for a review with many references). Here, two parallel branes approach each other, collide, and then rebound, moving in one of the spatial dimensions available. The big bang occurs as a bounce periodically an infinite number of times, each time there is a collision, and then there is a cyclic process of contraction, bounce and expansion, like in the old Tolman oscillating universe. There is a stage of early acceleration in this model that keeps the entropy from diverging in the cycle-to-cycle evolution after many cycles, and according to the authors there are no infinities in the defining quantities like in the standard cosmological model. The ekpyrotic model is but one of many possible bouncing cosmological models (see [113] for a related review).

Braneworld models nowadays abound. They have different motivations than the original brane models, but they all show the richness of brane cosmological ideas. Two such models are briefly noted below, one motivated by an attempt for a successful resolution of the cosmological constant problem, and the other by an adaptation of holographic ideas in cosmology. For a resolution of the λ problem in a higher-dimensional, brane setting, one uses a self-tuning mechanism and considers a single 3-brane universe in a five-dimensional bulk space with *linear or nonlinear bulk-fluids* (cf. [114] and related references therein). The main question here is whether there are regular solutions that satisfy a number of plausible physical criteria (finite Planck mass, energy conditions, etc.). In these braneworld models, ‘everything depends on the extra transverse Y-coordinate’, grossly violating the cylinder condition met in the original Kaluza–Klein universes. On the other hand, a particularly interesting feature of these models is the fact that a Wick-type of exchange between the time and the transverse spatial coordinates leads to cosmological models with many features having brane-theoretic origins.

The last brane model we discuss is *ambient cosmology*. This is an attempt to relate holographic techniques [115] in a braneworld setting with methods of conformal geometry to motivate the idea that the brane representing ‘our world’ has moved to the conformal infinity of the bulk, the ‘ambient space’. The resulting *ambient cosmology* has a number of novel characteristics that allow for previous problems such as the singularity problem and the question of cosmic censorship to be resolved very smoothly in this set-up [116].

In conclusion, braneworld cosmology presents interesting, well-posed problems for the future in terms of challenging the predictions of inflation as viable alternatives, as well as a variety of mathematical issues, which, in the face of the great number of unexplored possibilities in this part of cosmological model building, become particularly attractive to tackle.

### (c) Measures in the landscape

Interestingly, the picture of the inflationary multiverse according to eternal inflation and developed in the period 1980–2000, was found to be strongly supported by a string- or M-theoretic prediction of a huge collection of allowed, or even predicted, different vacua known as *the landscape* [117].

Conversely, eternal inflation provides a mechanism to ‘populate the landscape’ [118], another being that of the sum-over-histories (no-boundary) approach [119,120].

These two versions of a multiverse are part of a broader classification given in [121], where many other speculative versions of the idea of a multiverse as discussed.

An open question here is in what sense all these different ‘universes’ are really distinct to each other and how one would be able to avoid counting same ones as different. The general problem of how such a structure may evolve is also unknown, but of course no one really knows the nature of what holds the multiverse together, or how M-theory operates in this ‘moduli space of supersymmetric vacua’ (to quote [117]). This is one of the frontiers of theoretical mathematical cosmology.

### (d) Topology and cosmology

Another quantum possibility (or rather more like a speculation at the time) was suggested in 1973 by Tryon [122]. He imagined the universe as a quantum fluctuation of the vacuum, appearing accidentally so to speak ‘from time to time’ and obeying Heisenberg’s uncertainty principle. Thus an infinite lifetime would be associated with zero energy, but the unexplained issue with this proposal was why the universe had such a large age. But what would be the properties of a universe created from a fluctuation of the vacuum? A finitely born universe would have all possible shapes, and the Einstein equations only relate the local properties of space–time to the overall matter density. The issue of *topology of the universe* relates to global properties, possibly observable and in fact something like this could alter the images of observed galaxies by producing multiple, fainter copies of their images.

Sokolov & Shvartsman [123] and Gott [124] gave lower bounds on the size of a finite universe. Also Zeldovich & Starobinski [125] studied a flat, finite, universe as a quantum fluctuation and concluded that it must have an approximate spherical shape to avoid a singularity. Another issue with all these studies was how to create an infinite model with global non-trivial topology. It is difficult not to take seriously any model that allows for some mild form of non-trivial global topology. Today the problem of observationally detecting some aspect of large-scale topology is an active one [126].

As we discuss in more detail in §3f, the number of arbitrary spatial functions to describe the generality of a cosmology is four in vacuum for Einstein’s gravity and becomes 16 for higher-order gravity. This in turns becomes 4(1+F)+2S and 16+4F+2S, respectively, when a number F of fluids and a number S of scalar fields is added, cf. [127] (this counting assumes that dark energy is described by a cosmological constant).

It is an interesting result, which we now discuss that in the case of a Bianchi (homogeneous) cosmology having compact spatial topology—where because of the homogeneity assumption the arbitrary functions become constants—the corresponding numbers of constants require to determine the general solution generally increase without bound. This constitutes a major difference with respect to the corresponding situation of ‘trivial’ topologies. In the general inhomogeneous case, the situation is largely unknown.

The Einstein equations do not impose any restrictions on the spatial topological complexity of a solution, and this means that one may arbitrarily change the spatial topology of an original space–time-solution of the field equations and still keep the resulting configuration as a solution. Topological complexity, that is the complexity of a given topology of some solution to the field equations, is something that is measured by the number of the moduli degrees of freedom, namely, the number $N_{M}=6g+2k-6$, where g denotes the genus and k the number of conical singularities of the underlying orbifold. (Although every manifold is trivially an orbifold, the converse is not true. To get an idea of what an orbifold very roughly looks like, take a properly discontinuous action of a group Γ acting on a manifold M, then an orbifold looks locally like the coordinate system chart M→M/Γ. It is an orbit-manifold.)

Returning to our discussion of Bianchi spatial topologies, it is an intriguing fact that when compact spatial topologies are admitted to the standard Bianchi universes, the well-known properties met in standard Bianchi cosmology change to such a degree that the situation becomes almost reversed (see [128–134] for more information). Some Bianchi universes (IV and VI${\;}_{h}$) no longer exist, while those that contained the FRWL universes and were the generic ones with trivial topologies, now are no longer generic. For example, the VII${\;}_{h}$ universe must be isotropic and hence not generic, and the Bianchi IX universe with compact topology of any complexity is non-generic, but B-III and B-VIII now become the most general types, although they do not contain FRWL universes as special cases and get arbitrarily close to them.

The conclusion of the consideration of anisotropic universes with compact topologies is that they become heavily restricted, while isotropic ones are non-generic. Compact open universes become necessarily isotropic if they are assumed homogeneous, and flat spaces are generally preferred than closed or open ones.

The consideration of inhomogeneities and/or higher dimensionality may lead to further restrictions in future cosmological theories that may not be obtained otherwise except by such topological considerations. This is because there is an intimate relationship between inhomogeneity on a cosmologically large scale and topology [135]. We recall that the degree of a manifold, degM, equal to the dimension of its isometry group, is a number that shows the generality of it, and for compact manifolds (like the ones we have been considering in this section) degM=0 if and only if there is no non-zero global Killing field. Now according to Bochner’s theorem, compact manifolds having negative Ricci curvature admit no non-zero global Killing vectorfields, which in turn implies that they can only be *locally* homogeneous. We note that from local isotropy (say about us) one can only conclude *local* homogeneity (because of constant curvature), not a global one as it is usually assumed in the Bianchi class.

The broader class of locally homogeneous cosmologies becomes therefore a very interesting area of study in mathematical cosmology (cf. [136] and references therein). This subject closely ties and hugely extends the area of Bianchi topology through an analysis of Hamiltonian cosmology in this context. The results are truly remarkable and lead to a completely new picture of what topological structures may arise in a global situation under the Einstein evolution. A pivotal role is played by the mathematical theory of symplectic reduction giving in the present context a reduced Hamiltonian function for cosmology, such that asymptotically in the future direction of cosmological expansion the evolution is dominated by the so-called hyperbolizable components, on each one of which the conformal geometry described by a suitable metric becomes homogenous and isotropic, locally indistinguishable from a negatively curved FRWL domain, cf. [137] for a review and [138] for more recent work on this problem.

Understanding the global behaviour of solutions to any set of cosmological equations (such as the Einstein equations) and their dependence upon changes of spatial topology is one of the most important problems of mathematical cosmology.

### (e) Dynamical singularities

As we have already indicated, the nature of cosmological singularities is a problem left out by the singularity theorems of general relativity, or by the corresponding ones in eternal inflation. The issue of the nature of singularities is indeed a very complex one, having many facets, and has recently received a further boost of activity, which we shall now briefly discuss.

There is a classification of homogeneous universes reviewed in Collins & Ellis [139] exploiting techniques used in the singularity theorems (e.g. the Raychaudhuri equation) as well as qualitative studies of the field equations themselves (for the purposes of this classification, the field equations are ordinary differential equations so that standard dynamical systems techniques may be applied). In this case, there arise infinite density singularities as well as infinities in certain curvature invariants in all FRWL and orthogonal Bianchi universes. An interesting aspect of this classification is, however, that the big bang is not necessarily a point singularity, but other types are possible, such as pancakes, cigars, etc.

The situation changes completely in tilted Bianchi models (except type IX) where, apart from big bang singularities, there are now finite density ones, where there is the intriguing possibility that the fluid flow may continue past the finite density singularity, as the flow lines turn null and a Cauchy horizon is developed where the evolutions comes to an end (cf. figure 1, and Section 8 of [139]). This behaviour may be very clearly seen in type V models (which contain the open FRWL universes), and the overall behaviour is very different from that occurring in the simplest isotropic models for a variety of fluids. For Bianchi-type IX universes instead, we have the closed universe recollapse theorem of R. M. Wald, which states that there do not exist any eternally expanding Bianchi IX universes with matter satisfying the dominant energy condition, and has non-negative average pressure [140].

There is however another type of singularity classification that is based on the work of Choquet–Bruhat and stems from theorems giving sufficient conditions for causal g-completeness, that is necessary conditions for the development of cosmological singularities (cf. [141], [142, ch. 8]). This leads directly to a complete dynamical classification for isotropic cosmologies into many distinct types [143,144]. In fact, there are four types of this classification that are found to play an important role in current models, as they appear commonly in dark energy universes with phantom fluids [145].

This leads to a ‘zoo’ of cosmological singularities developing in finite time into the future, already in the isotropic category, to say nothing about possibilities in more general homogeneous universes. These singularities include not only the traditional ones discussed earlier (big bang and big crunch) but many other types, such as big rips, sudden singularities, or very mild soft ones, turnarounds, etc. (see [146] for a brief review). They can be studied by using generalized power series [147,148], or the method of dominant balance [149], and further classified using the notion of strength of singularities. These studies have provided a clear picture of the dynamical behaviours possible, but are limited only in the isotropic category.

### (f) Generic universes

As we have already discussed earlier, a common property of most of the cosmological solutions met in Einstein’s theory is the occurrence of infinite curvatures and densities a finite time in their past, a ‘cosmological singularity’. Despite lacking a rigorous definition of what a singularity is at the time, Lifshitz and Khalatnikov in 1963 (LK hereafter) were able to show that the *generic singularity* present in typical solutions of the Einstein equations cannot have the usual simple, monotone, power-law character predicted by isotropic (or simple anisotropic) solutions [150]. Their analysis also extended the Lifshitz 1946 instability study to the more general situation where the examined perturbations were now comparable with the horizon scale (proportional to the scale factor in a Friedmann universe).

In their analysis, LK introduced a method of proof that was based on series expansions around some unperturbed state (vacuum, or simple radiation solutions), and then counting the arbitrary functions present in the resulting solution of the field equations considered in the synchronous reference system. If that number was equal to the maximum allowed number by the initial value problem, they concluded that the solution was a general one, otherwise the solution was a special one based on a smaller number of free initial data (cf. §3d). For instance, the number of free initial data required for a general solution is equal to 4 for vacuum general relativity.

This function counting method is more useful than it is expected naively. There are solutions with the required number of arbitrary functions (in terms of function counting) to quantify as general solutions of the field equations of a given theory, and others that have smaller such numbers. In the latter case, the solutions possess a ‘transient’ nature, they are unstable. In the former case, they are general solutions of the field equations, and consequently stable in the time intervals they are taken. For example, full functions counting solutions include the perturbed de Sitter space found in [151], the sudden finite-time singularity solution in general relativity [152], and in Brans–Dicke theory [153], and the ultrastiff perfect fluid solution having p>ρ near quasi-isotropic singularities [154]. On the other hand, unstable, transient solutions are more common, for example, the standard quasi-isotropic solutions in general relativity is unstable to perturbations within that theory, cf. [155, p. 368], [156,157], and so are solutions containing one or more fluids in that theory [158,159]. For other approaches to the function counting problem, see [160,161] and references therein.

The functions counted in these problems have simple physical interpretations. For example, the four free parameters in general relativity specifying a general solution in vacuum describe two shear modes plus anisotropies in the spatial curvature. When matter is added, the extra functions are the density or pressure, and the three (non-comoving) fluid velocity components, and although these contribute to the temperature fluctuations of the CMB radiation, they can be usually ignored being too small to be detected presently. However, many of the more elaborate cosmological models discussed in this paper, e.g. eternal inflation or M-theoretic models, predict effects that are in principle described by many arbitrary functions of the sort appearing in function counting problems. For inflation, they are also unobservable lying beyond our particle horizon, albeit isotropic.

### (g) Dark energy

*Dark energy* representing the major component of the current acceleration of the universe has become the prime focus of attention since 1998 when two groups [162,163] using different datasets and techniques discovered that observations showed that the speed of the expansion of distant supernovae curved upwards with relation to their distance. This discovery has had an immense impact on cosmology ever since, and confirmed the expectations of a cosmological constant—the so-called ΛCDM (CDM here stands for ‘cold dark matter’, cf. [164, Section 7.2]) phenomenological model—but allowed for the existence of many more exotic stresses to describe this mysterious kind of energy.

Today models of dark energy span the whole range of possibilities, from a cosmological constant, to scalar fields—the so-called quintessence—to modified gravity theories. It is indeed an amazing feature of this hypothetical type of energy the fact that it has unified in a single quest the incredible spectrum of modified gravity theories and other exotic forms of energy to search for the missing explanation of this observed effect.

Dark energy appears as a property inherent in space–time because it is perfectly uniform and insensitive to space–time being empty or full of galaxies, or with respect to the direction we look, or the era in the universe history, or spatial location. The constraints from the supernovae observations, the CMB temperature fluctuations and the baryon acoustic oscillations (that is residual sound waves imprinted in the clustering patterns) all converge to the astonishing dark energy budget of about 72% in the overall matter energy content of the universe. Another component of the remaining material in the universe consists of dark matter with a percentage of about another 24%. The mysterious fact that the visible components of matter and radiation may constitute only about a tiny 4% of the overall distribution, represents perhaps the most unbelievable, unexplained result in the whole history of astronomy.

We thus see that the fundamental themes required for an adequate explanation of the cosmological constant, the energy of the vacuum and the nature of dark energy are all linked together as suggested by both theory and observations. The completely and seemingly disparate and independent fields such as scalar-tensor theory, higher-order gravity, theories with screening, Palatini cosmology, effective theories, Horndeski gravity, higher-dimensional cosmology, Born–Infeld type theories, holographic theory and many others, appear as one field having different facets in this sense; for various excellent reviews of this vast field, see [165–172].

## 4. Contents and abstracts of Part B of the Theme Issue

The abstracts of the contributions to volume 2 of the Theme Issue are as follows.

(i) Brandenberger [173], Limitations of an effective field theory treatment of early universe cosmology
  Assuming that superstring theory is the fundamental theory which unifies all forces of Nature at the quantum level, I argue that there are key limitations on the applicability of effective field theory techniques in describing early universe cosmology.
(ii) Gasperini [174], From pre- to post-big bang: an (almost) self-dual cosmological history
  We present a short introduction to a non-standard cosmological scenario motivated by the duality symmetries of string theory, in which the big bang singularity is replaced with a ‘big bounce’ at high but finite curvature. The bouncing epoch is prepared by a long (possibly infinitely extended) phase of cosmic evolution, starting from an initial state asymptotically approaching the string perturbative vacuum.
(iii) Antoniadis *et al.* [175], Brane-world singularities and asymptotics in a five-dimensional fluid bulk
  We review studies on the singularity structure and asymptotic analysis of a 3-brane (flat or curved) embedded in a five-dimensional bulk filled with a ‘perfect fluid’ with an equation of state p=γρ, where p is the ‘pressure’ and ρ is the ‘density’ of the fluid, depending on the fifth space coordinate.
  Regular solutions satisfying positive energy conditions in the bulk exist only in the cases of a flat brane for γ=−1 or of AdS branes for γ∈[−1,−1/2). More cases can be found by gluing two regular brunches of solutions at the position of the brane. However, only a flat brane for γ=−1 leads to finite Planck mass on the brane and thus localizes gravity. In a more recent work, we showed that a way to rectify the previous findings and obtain a solution for a flat brane and a range of γ, that is both free from finite-distance singularities and compatible with the physical conditions of energy and finiteness of four-dimensional Planck mass, is by introducing a bulk fluid component that satisfies a nonlinear equation of state of the form $p=\gamma \rho^{\lambda }$ with γ<0 and λ>1.
(iv) Vanhove [176], A S-matrix approach to gravitational waves physics
  The observation of gravitational waves emitted by binary systems has opened a new astronomical window into the Universe. We describe recent advances in the field of scattering amplitudes applied to the post-Minkowskian expansion, and the extraction of the effective two-body gravitational potential. The techniques presented here apply to any effective field theory of gravity and are not restricted to four-dimensional Einstein gravity.
(v) Sola [177], Cosmological constant problem and running vacuum in the expanding universe
  It is well known that quantum field theory (QFT) induces a huge value of the cosmological constant, Λ, which is outrageously inconsistent with cosmological observations. We review here some aspects of this fundamental theoretical conundrum (the cosmological constant problem) and strongly argue in favour of the possibility that the cosmic vacuum density $\rho_{\mathrm{vac}}$ may be mildly evolving with the expansion rate H. Such a ‘running vacuum model’ (RVM) proposal predicts an effective dynamical dark energy without postulating new ad hoc fields (quintessence and the like). Using the method of adiabatic renormalization within QFT in curved space–time we find that $\rho_{\mathrm{vac}}(H)$ acquires a dynamical component $O(H^{2})$ caused by the quantum matter effects. There are also $O(H^{n})$ (n=4,6,…) contributions, some of which may trigger inflation in the early universe. Remarkably, the evolution of the adiabatically renormalized $\rho_{\mathrm{vac}}(H)$ is not affected by dangerous terms proportional to the quartic power of the masses ($∼m^{4}$) of the fields. Traditionally, these terms have been the main source of trouble as they are responsible for the extreme fine tuning feature of the cosmological constant problem. In the context under study, however, $\rho_{\mathrm{vac}}(H)$ is currently dominated by a constant term plus the aforementioned mild dynamical component $∼\nu H^{2}$ (|ν|≪1), which makes the RVM to mimic quintessence.
(vi) Erickson *et al.* [178], Higgs effect without lunch
  Reduction in effective space–time dimensionality can occur in field-theory models more general than the widely studied dimensional reductions based on technically consistent truncations. Situations where wave function factors depend non-trivially on coordinates transverse to the effective lower dimension can give rise to unusual patterns of gauge symmetry breaking. Leading-order gauge modes can be left massless, but naturally occurring Stueckelberg modes can couple importantly at quartic order and higher, thus generating a ‘covert’ pattern of gauge symmetry breaking. Such a situation is illustrated in a five-dimensional model of scalar electrodynamics in which one spatial dimension is taken to be an interval with Dirichlet/Robin boundary conditions on opposing ends. This simple model illuminates a mechanism which also has been found in gravitational braneworld scenarios.
(vii) Townsend [179], Aether, dark energy, and string compactifications
  The nineteenth century Aether died with Special Relativity but was resurrected by General Relativity in the form of dark energy; a tensile material with tension equal to its energy density. Such a material is provided by the D-branes of string-theory; these can support the fields of supersymmetric particle-physics, although their en-energy density is cancelled by orientifold singularities upon compactification. Dark energy can still arise from supersymmetry-breaking anti-D-branes but it is probably time-dependent. Recent results on time-dependent compactifications to an FLRW universe with late-time accelerated expansion are reviewed.
(viii) Tan *et al.* [180], How inflationary gravitons affect gravitational radiation
  We include the single graviton loop contribution to the linearized Einstein equation. Explicit results are obtained for one loop corrections to the propagation of gravitational radiation. Although suppressed by a minuscule loop-counting parameter, these corrections are enhanced by the square of the number of inflationary e-foldings. One consequence is that perturbation theory breaks down for a very long epoch of primordial inflation. Another consequence is that the one loop correction to the tensor power spectrum might be observable, in the far future, after the full development of 21 cm cosmology.
(ix) Kazanas *et al.* [181]. Gravity beyond Einstein? Yes, but in which direction?
  We present qualitative arguments in favour of an extension of the theory of the gravitational interaction beyond that resulting from the Hilbert–Einstein action. To this end, we consider a locally conformal invariant theory of gravity, discussed some 30 years ago by Mannheim and Kazanas. We discuss its exact solution of the static, spherically symmetric configurations and, based on these, we revisit some of the outstanding problems associated with gravity, high energy interactions and sketch potential resolutions within the conformal gravity framework.
(x) Dadhich [182], On space–time structure and the Universe: some issues of concept and principle
  In this discourse, we would like to discuss some issues of concept and principle in the context of the following three aspects. One, how Λ arises as a constant of space–time structure on the same footing as the velocity of light. These are the two constants innate to space–time without reference to any force or dynamics whatsoever, and are interwoven in the geometry of ‘free’ homogeneous space–time. Two, how does vacuum energy gravitate? Could its gravitational interaction in principle be included in general relativity or a new theory of quantum space–time/gravity would be required? Finally, we would like to raise the fundamental question, how does physically the Universe expand? Since there does not lie anything outside, it cannot expand into, instead it has to expand of its own—may be by creating new space out of nothing at each instant! Thus not only was the Universe created at some instant in the past marking the beginning in the big-bang, and at its edge it is also continuously being created at each epoch as space expands. We thus need quantum theory of space–time/gravity at the both ends—ultraviolet as well as infrared.

## Data Availability

This article has no additional data.

## Footnotes

1 In distinction to the case of the BD, or scalar-tensor, theory, this scalar field was defined as proportional to the $\ln f^{\prime }(R)$. Although there are claims in the literature of a relation of f(R)-gravity to Brans–Dicke theory by pure redefinition of the fields, these are based on non-cosmological scalar fields (such as proportional to the scalar curvature thus becoming negligible at cosmological scales), and extra conditions like $f^{\prime \prime }(R)\neq 0$, which unnecessarily restrict the original theory. On the other hand, conformal equivalence does not require such relationship between the two classes of theory, and shows that both f(R)-gravity and Brans–Dicke type theories are *directly* conformally equivalent to GR plus suitable scalar fields.
